# Identifying predictors for postoperative clinical outcome in lumbar spinal stenosis patients using smart-shoe technology

**DOI:** 10.1186/s12984-017-0288-0

**Published:** 2017-07-18

**Authors:** Sunghoon I. Lee, Andrew Campion, Alex Huang, Eunjeong Park, Jordan H. Garst, Nima Jahanforouz, Marie Espinal, Tiffany Siero, Sophie Pollack, Marwa Afridi, Meelod Daneshvar, Saif Ghias, Majid Sarrafzadeh, Daniel C. Lu

**Affiliations:** 1 College of Information and Computer Science, UMass Amherst, Amherst, USA; 2 Neuroplasticity and Repair Laboratory, UCLA, Los Angeles, USA; 3 Neuromotor Recovery and Rehabilitation Center, UCLA, Los Angeles, USA; 4 Department of Neurosurgery, UCLA, Los Angeles, USA; 5 Cardiovascular Research Institute, Yonsei University College of Medicine, Los Angeles, USA; 6 Computer Science Department, UCLA, Los Angeles, USA; 7 Department of Orthopaedic Surgery, UCLA, Los Angeles, USA

**Keywords:** Lumbar spinal stenosis, Smart shoes, Walking test, Prediction, Oswestry disability index, Predominant pain

## Abstract

**Background:**

Approximately 33% of the patients with lumbar spinal stenosis (LSS) who undergo surgery are not satisfied with their postoperative clinical outcomes. Therefore, identifying predictors for postoperative outcome and groups of patients who will benefit from the surgical intervention is of significant clinical benefit. However, many of the studied predictors to date suffer from subjective recall bias, lack fine digital measures, and yield poor correlation to outcomes.

**Methods:**

This study utilized smart-shoes to capture gait parameters extracted preoperatively during a 10 m self-paced walking test, which was hypothesized to provide objective, digital measurements regarding the level of gait impairment caused by LSS symptoms, with the goal of predicting postoperative outcomes in a cohort of LSS patients who received lumbar decompression and/or fusion surgery. The Oswestry Disability Index (ODI) and predominant pain level measured via the Visual Analogue Scale (VAS) were used as the postoperative clinical outcome variables.

**Results:**

The gait parameters extracted from the smart-shoes made statistically significant predictions of the postoperative improvement in ODI (RMSE =0.13, *r*=0.93, and *p*<3.92×10^−7^) and predominant pain level (RMSE =0.19, *r*=0.83, and *p*<1.28×10^−4^). Additionally, the gait parameters produced greater prediction accuracy compared to the clinical variables that had been previously investigated.

**Conclusions:**

The reported results herein support the hypothesis that the measurement of gait characteristics by our smart-shoe system can provide accurate predictions of the surgical outcomes, assisting clinicians in identifying which LSS patient population can benefit from the surgical intervention and optimize treatment strategies.

**Electronic supplementary material:**

The online version of this article (doi:10.1186/s12984-017-0288-0) contains supplementary material, which is available to authorized users.

## Background

Lumbar Spinal Stenosis (LSS) is a chronic spinal condition most commonly experienced by individuals over 50 years of age [1]. It is estimated that approximately 2.4 million individuals in the United States, 8−11*%* of the country’s population, will be affected by LSS by 2021 [2]. Lumbar decompression surgery is the most effective and frequently utilized surgical intervention strategy. However, approximately one third of the patients who undergo surgery are not satisfied with their postoperative outcomes due to pain and inferior functional level [3, 4]. Given these findings, identifying preoperative predictors of surgical outcomes, which is referred to as outcome research, would be of significant clinical utility as it allows healthcare providers to define realistic and achievable postoperative goals for patients and their caregivers [3, 5–7]. Furthermore, a prediction algorithm can prevent patients who may not benefit from a surgical intervention from receiving such an invasive and costly treatment.

Outcome research, with the general goal of improving care to promote the best interests of the patient, can take on many forms depending on what the final outcome of interest is [8, 9]. Regarding LSS specifically, numerous studies have investigated a wide spectrum of preoperative clinical variables in order to predict postoperative outcomes of disability, health, and quality of life. Measurement tools to quantify these outcomes have included health-related quality of life measure (HRLQoL), Oswestry Disability Index (ODI), Short Form 36 (SF-36), Roland-Morris (RM) questionnaire, Core Outcomes Measures Index (COMI), and pain level [3, 10–13]. Using the ODI at two-year follow-up, Aalto et al. found regular preoperative analgesic use, non-smoking status, and above-average self-rated health as statistically significant outcome predictors [10]. Similarly, Sanden et al. used the ODI, SF-36, and EuroQol questionnaires at two-year follow-up to support preoperative smoking status as a predictor of poorer outcomes [11]. Use of the modified RM questionnaire by Athiviraham et al., at these same time intervals, found a higher BMI and a history of psychiatric disease to be predictors of poorer outcome [12]. Sigmundsson et al. utilized the HRLQoL and ODI to show that the duration of leg pain prior to surgical intervention serves as an outcome predictor at a one-year postoperative follow-up interval [3]. Finally, use of the COMI by Sobottke et al. demonstrated that having a history of fewer previous surgeries, rigid or dynamic stabilization, and lower patient comorbidity, were all predictors of improved outcome [13]. However, despite the statistical significance, most of the aforementioned work did not provide great predictability of postoperative outcomes. More importantly, they focused on finding predictors from clinical variables that were available in the hospital database system, rather than variables that could directly quantify the patients’ neurological condition relevant to the functional deficits that surgery attempts to address (e.g., walking ability).

Recently, researchers and clinicians have started to investigate walking ability as a predictor in LSS patients [14–16]. These studies focused on walking capacity, which is defined as the distance a person is able to walk continuously on a flat surface at a self-selected pace until being forced to stop due to symptoms of LSS, up to a limit of 30 min. The walking capacity and total walking duration during the preoperative visit showed significant correlations to postoperative ODI scores [14–16]. However, given that measurements of walking capacity can take up to 30 min, this protocol has limitations in the busy clinical setting and poses a significant physical burden on patients. As a result, various neurological and physical exam maneuvers have been investigated as possible supplements, but none to date have functioned as predictors of surgical outcomes [6, 7].

The proposed study presents a novel technological approach that utilizes a pair of sensorized smart-shoes during a preoperative 10 m walking test to predict functional outcomes following nerve root decompressive surgery in LSS patients. Two postoperative outcomes, namely the ODI and predominant pain level measured by the Visual Analogue Scale (VAS), were used in this study. The smart-shoe was equipped with an array of five pressure sensors on the insole, which allowed to extract comprehensive analysis of spatiotemporal gait characteristics. More specifically, this paper first demonstrates that these preoperative gait parameters obtained from a 10 m walking test can better predict the postoperative outcomes when compared to conventional clinical variables that were previously studied. Then, this paper further demonstrates that even more accurate predictions can be achieved by combining the gait parameters with clinical variables using the linear regression algorithm.

## Methods

### Patients

A total of 29 LSS patients (21 female) with ages ranging from 59.1±15.9, were recruited from the UCLA Spine Center. Inclusion criteria entailed diagnosis of LSS (lumbar disk herniation, lumbar spondylolisthesis, and/or adjacent segment disease) with radiculopathy and/or axial pain in the lower limbs that affected their walking ability. Diagnosis of LSS was verified using Magnetic Resonance Imaging (MRI). Patients with other neuromuscular or spinal cord conditions were excluded. All patients underwent lumbar decompression (laminotomy, foraminotomy, discectomy) and/or lumbar fusion surgery, performed by a single neurosurgeon (DCL). Fifteen of these patients (11 female) with ages ranging from 58.4±16.8, agreed to be reevaluated at least three months after the surgical intervention. The experimental procedure was approved by the UCLA institutional review board (IRB# 12-000009), and all patients provided consent to participate in the study.

### Walking test procedure

**Fig. 1:**
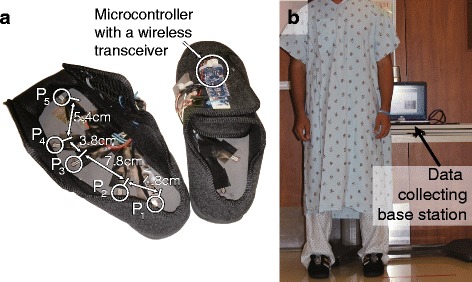
**a** The *sensorized smart-shoes* platform containing *five pressure sensors* (P_1_ to P_5_) capture spatiotemporal gait characteristics, and a microcontroller and wireless transceiver to transmit the captured data to the base station (*laptop*). **b** An individual wearing the platform for a walking test in the clinical setting

During the preoperative visit, patients were asked to perform a 10 m self-paced walking test (SPWT) while wearing a pair of sensorized smart-shoes developed at the UCLA Wireless Health Institute. Figure 1 illustrates the smart-shoes that were used in the experiment. The smart-shoes were equipped with an array of five pressure sensors (FSR400, Interlink Electronics, USA) that have been widely used to capture kinetic parameters of gait [17, 18]. A microcontroller (Fio, Arduino, Italy) was employed to sample the pressure data at 50 Hz and wirelessly transmit to a base station (i.e. a laptop) via the IEEE 802.15.4 (XBee) standard protocol. The pressure sensors were positioned at locations that help elucidate spatiotemporal characteristics of patients’ walking patterns: P_1_ to detect heel-strike, P_3_ to detect mid-lateral plantar pressure, P_5_ to detect toe-off, and P_2_ and P_4_ to detect weight distribution between the aforementioned three locations. A total of ten pairs of different sized shoes were made (five for males and five for females), and the pressure sensor locations were placed linearly proportional to each other. Three different female (U.S. size 6, 7, and 8.5) and two male (U.S. size 7 and 11) shoes were used in this experiment.

**Fig. 2:**
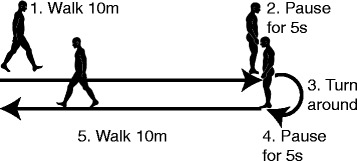
Illustration of the 10 m walking test performed in this work. Patients were asked to walk at their preferred speed on a 10 m trail, pause for five seconds, turn around, pause for another five seconds, and walk back along the 10 m trail to the initial position

Patients were asked to walk at their preferred speed on a 10 m trail that was marked in the hospital hallway. Then, they were asked to pause for five seconds, turn around, pause for another five seconds, and walk back along the 10 m trail to the initial position as shown in Fig. 2. The pauses between walking and turning were utilized to segment the walking data. Patients repeated the above procedure twice, producing a total of four 10 m walks.

### Independent variables: gait parameters and clinical variables

The obtained sensor data produced a total of 39 spatiotemporal measurements representing various dimensions of gait characteristics in LSS patients. In this section, we elaborate on a subset of important gait measurements that we believe are worth noting. Detailed information for all 39 measurements is provided in the Additional file 1: Appendix.

MeanTime-P _*i*_ *P*_*j*_ and StdDevTime-P _*i*_ *P*_*j*_ represent the mean and standard deviation of the time difference between the peaks of P _*i*_ and P _*j*_, respectively. The computed time difference was normalized to the shoe size (i.e. length of the shoe) in order to remove distance-dependent variability. These parameters quantify how quickly and consistently a subject distributes his/her weight on the insole of the foot during walking. For example, MeanTime-P_2_P_3_ computes the time taken to shift the weight from the proximal to distal regions of the lateral side of the foot. AutoCorr-P _*i*_ computes the maximum auto-correlation of the time series of P _*i*_, which quantifies how consistently the subject distributed his/her weight to the pressure sensor P _*i*_ during walking. SumMag-P _*i*_ and StdDevMag-P _*i*_ respectively represent the sum and standard deviation of the amplitudes of P _*i*_, which similarly quantify the consistency of pressure applied to P _*i*_ during gait. These parameters, except AutoCorr-P _*i*_, were computed per gait cycle on each foot. Then, the measurements were averaged over the gait cycles that were obtained from the four 10 m walks. The value of AutoCorr-P _*i*_ was computed per 10 m walk and averaged out of the four walks. This resulted in two measurements from the two feet, one from each foot. Then, the minimum, maximum, and mean of the two values were considered in this work in order to emphasize the unilateral (minimum and maximum), and bilateral (mean) characteristics of the motor symptoms in LSS patients. For example, MeanTime-P _*i*_ *P*_*j*_-Max, MeanTime-P _*i*_ *P*_*j*_-Min, and MeanTime-P _*i*_ *P*_*j*_-Mean represent the maximum, minimum and mean of the MeanTime-P _*i*_ *P*_*j*_ values of the two feet. This study also included some gait measurements that were computed as a function of sensor data from the two shoes. The symmetry index of gait, SymIndex, investigated the bilateral symmetry between the two limbs as introduced in [19]: SymIndex = $(T_{R} - T_{L})/\frac {1}{2}(T_{R} + T_{L})$, where *T*_*R*_ and *T*_*L*_ represent the average stride time for left and right feet, respectively. CrossCorr-P _*i*_ computed the maximum cross-correlation of the time series of P _*i*_ of the left and right shoes, which quantified the similarity of the gait pattern between the two limbs. Table 1 summarizes the gait measurements considered in this work.

**Table 1:** Description of the important gait measurements that were considered in this work

Name	Description
MeanTime-P _*i*_ *P*_*j*_	The mean of the time different between the peaks of P _*i*_ and P _*j*_.
StdDevTime-P _*i*_ *P*_*j*_	The standard deviation of the time different between the peaks of P _*i*_ and P _*j*_.
AutoCorr-P _*i*_	The maximum auto-correlation of the time series of P _*i*_.
SumMag-P _*i*_	The sum of the amplitudes of P _*i*_.
StdDevMag-P _*i*_	The standard deviation of the amplitudes of P _*i*_.
SymIndex	The bilateral symmetry between the two limbs as introduced in [19].
CrossCorr-P _*i*_	The maximum cross-correlation of the time series of P _*i*_ of the left and right shoes

A total of 10 clinical variables that were previously found to be predictive of postoperative outcome in LSS patients were also considered. These variables include age [7, 20], gender [6, 7, 20], self-rated walking ability [6], presence of scoliosis [3, 7], presence of acute injury, number of affected spinal vertebrae, number of previous spinal surgeries [13], duration of symptoms [3], BMI [12], and smoking history [11]. Furthermore, the preoperative evaluations of the patient-reported functional measurements were also considered as predictors [13].

### Dependent variables: patient-reported functional outcomes

Two dependent variables were considered in this work: the ODI [21] and predominant pain level measured by the VAS [3] that were reported by the patients postoperatively. The ODI is one of the most commonly used clinical measures to evaluate low-back disability [3, 22]. The ODI consists of ten questions concerning intensity of pain and the degree of disability in performing activities of daily living (ADL), such as sleeping, self-care, sex life, social life, and traveling [21]. is scored out of five or six, with zero indicating the least amount of disability. Patients must check the statement that most closely resembles their functional status. The accumulated score is linearly scaled from 0 (no disability) to 100 (total disability) [21]. The ODI was administered at the baseline and follow-up visits; note that the postoperative ODI score was used as the dependent variable. The predominant symptom (either lower back or leg pain) was also evaluated pre and postoperatively using VAS. Patients evaluated their pain level by drawing a position along a continuous line between two end-points indication no pain and unbearable pain. The pain level was quantified by ratio between the length of the patient-specified position from the no-pain end-point and the total length of the line, which was scaled from 0 (no pain) to 100 (unbearable pain). Note that the postoperative VAS score was used as another dependent variable.

Table 2 summarizes the patient demographics, clinical variables, some important spatiotemporal measurements from the smart-shoes, and the self-reported functional outcome values at both pre and postoperative visits.

**Table 2:** A summary of the patient demographics and self-reported functional outcomes (ODI and VAS)

		Baseline	Follow-up
		(*N*=15)	(*N*=17)
Patient demographics	Age	58.4±16.8	-
	Gender	11 female / 3 male	-
	Self-rated walking ability (0 to 3)	2.2±0.86	-
	Presence of scoliosis	1 yes / 14 no	-
	Presence of acute injury	4 yes / 11 no	-
	Number of affected spinal vertebrae	2.13±0.35	-
	Number of previous spinal surgeries	0.27±0.46 (4 subjects had 1, 11 had 0)	-
	Duration of symptoms	51.1±88.2 days	-
	BMI	26.3±5.04	-
	Smoking status	2 yes / 13 no	-
Important spatiotemporal measurements	StdTime-P_2_P_3_-Max	0.12±0.096	-
	CrossCorr-P_2_	4.2×10^4^±2.7×10^4^	-
	AutoCorr-P_2_-Mean	0.77±0.057	-
	SumMag-P_2_-Min	1.2×10^4^±5.7×10^3^	-
	MeanTime-P_2_P_3_-Mean	0.18±0.070	-
	AutoCorr-P_5_-Min	0.81±0.040	-
	SumMag-P_2_-Min	1.1×10^4^±5.7×10^3^	-
	MeanTime-P_1_P_2_-Max	0.18±0.15	-
Functional outcome	ODI	40.2±19.2	77.3±20.2
	VAS - Pain	49.9±34.5	33.4±31.9

### Analysis

This section presents the data analyses that investigated 1) weather the preoperative gait measurements would provide better predictability of surgical outcomes compared to the previously studied clinical variables, and 2) weather combining these gait and clinical variables would lead to more accurate prediction of surgical outcomes.

**Predictability of gait measurements compared to clinical variables:** Each (univariate) predictor candidate was compared to the two dependent variables using Spearman correlation [23, 24]. The correlation coefficient *r* and its *p*-value were used to evaluate the correlation. The value of *p*<0.05 was considered statistically significant.

**Multivariate prediction:** Multiple variables were combined together to predict the dependent variables. Multivariate linear regression was used to combine multiple predictors to predict the postoperative ODI and VAS. In order to avoid over-fitting of the prediction model to our relatively small number of data, the number of predictors used in the prediction model was limited as suggested in [25]; for a linear model, the ratio between the number of subjects and number of predictors was restricted to be 10:1. Since our dataset involved 15 patients with follow-up visits, a total of ⌊15/10⌉=2 predictors were utilized at a time. All possible combinations of two predictors were generated out of a total of 51 predictors (i.e. 39 spatiotemporal measurements from the smart-shoes, ten clinical variables, and two preoperative functional outcomes), and the combination that produced the largest *r* (i.e. strongest correlation) was reported. The prediction of postoperative outcome measure was made by combining the two selected variables via linear regression. The root mean squared error (RMSE) between the predicted and actual outcome measures was also reported.

## Results

### Predicting postoperative ODI

Each predictor candidate (both spatiotemporal measurements and clinical variables) was compared to the postoperative ODI score. Table 3 shows the correlation results of the top five spatiotemporal measurements with the largest absolute correlation coefficient values (i.e. |*r*|), all ten clinical variables, and the two functional outcomes (i.e. VAS and ODI) that were collected preoperatively. StdTime-P_2_P_3_-Max, which represents the standard deviation of the time between the peaks of P_2_ and P_3_ (the maximum value between the two feet), produced the highest absolute correlation (*r*=0.61) with *p*-value of 0.016. The clinical variable with the largest absolute correlation coefficient was smoking status, but the correlation was not statistically significant (*p*<0.13). Furthermore, the ODI that was collected preoperatively did not show significant correlation to the postoperative ODI (*p*<0.21).

**Table 3:** Correlations between the predictor candidates and postoperative ODI scores

Type	Predictors	*r*	*p*-value
Spatiotemporal measurements	StdTime-P_2_P_3_-Max	0.61	0.016
	CrossCorr-P_2_	−0.54	0.037
	AutoCorr-P_2_-Mean	−0.53	0.043
	SumMag-P_2_-Min	0.51	0.053
	MeanTime-P_2_P_3_-Mean	0.49	0.065
Clinical variables	Age	0.15	0.58
	Gender	0.077	0.78
	Duration of Symptoms	0.13	0.64
	Walking ability	−0.17	0.55
	Presence of scoliosis	0.31	0.26
	Smoking Status	0.41	0.13
	Acute Injury	−0.14	0.62
	Previous spine surgery	0.42	0.12
	Number of affected disk	−0.045	0.87
	BMI	0.36	0.18
Preoperative outcomes	Preoperative VAS	0.30	0.27
	Preoperative ODI	0.35	0.21

Top five spatiotemporal measurements with the largest absolute Spearman correlation coefficients (*r*) are listed. All clinical variables, including the functional outcomes that were collected preoperatively, are also listed

**Fig. 3:**
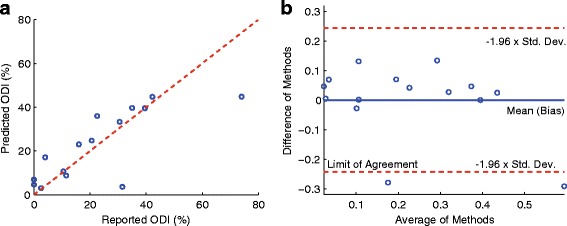
**a** *Scatter plot* between the reported ODI and predicted ODI scores, where the Spearman correlation coefficient *r* was 0.78, *p*<5.32×10^−4^, and RMSE of 0.13. **b** Bland-Altman plot where the bias was 2.41×10^−17^ and the limit of agreement was 0.24

**Fig. 4:**
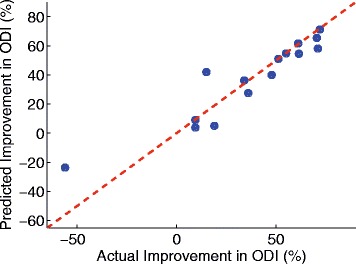
*Scatter plot* between the actual and predicted improvement in ODI after surgical operation. The Spearman correlation coefficient *r* was 0.93, *p*<3.82×10^−7^, and RMSE of 0.13

When two predictors were combined to predict the postoperative ODI score using multivariate linear regression technique, the combination of StdDevTime-P_2_P_3_-Max and SymIndex produced the strongest correlation coefficient of *r*=0.78 with *p*<5.32×10^−4^. The RMSE against the reported ODI value was 0.13. Figure 3 shows the scatter plot and the Bland-Altman plot. The bias of the difference between the predicted and actual ODI scores was 2.41×10^−17^, and the limit of agreement was 0.24. The predicted postoperative ODI score based on the multivariate analysis was subtracted from the preoperative score in order to predict the improvement of ODI after surgical operation. Figure 4 shows the statistically significant correlation between the predicted and actual improvement, which yielded the RMSE of 0.13 and Spearman correlation coefficient of *r*=0.93 with *p*<3.82×10^−7^. The effect of the unbalanced gender distribution in the sample (11 female vs. 4 male) on the estimation error was examined using an unpaired *t*-test, and did not show a statistically significant difference with *p*<0.55.

### Predicting postoperative VAS

Table 4 summarizes the correlation results between the postoperative VAS and individual predictors. Among all the considered possible predictors, the correlation results of the top five spatiotemporal measurements with the largest absolute correlation coefficients, all ten clinical variables, and the two preoperative functional evaluations (i.e. VAS and ODI) were reported. AutoCorr-P_2_-Mean, which represents the maximum autocorrelation of the time series of P_2_ (the mean of the feature values from the two feet), produced the highest correlation (*r*=−0.70) with *p*-value of 0.0035. None of the clinical variables and preoperative outcomes that were considered in this work produced significant correlation to postoperative VAS.

**Table 4:** Correlations between the predictor candidates and postoperative VAS scores

Type	Predictors	*r*	*p*-value
Spatiotemporal measurements	AutoCorr-P_2_-Mean	−0.70	0.0035
	CrossCorr-P_2_	−0.69	0.0044
	AutoCorr-P_5_-Min	−0.63	0.012
	SumMag-P_2_-Min	0.61	0.015
	MeanTime-P_1_P_2_-Max	0.60	0.017
Clinical variables	Age	0.33	0.22
	Gender	0.21	0.44
	Duration of symptoms	0.30	0.28
	Walking ability	−0.10	0.72
	Presence of scoliosis	0.12	0.66
	Smoking status	0.45	0.089
	Acute injury	−0.35	0.20
	Previous spine surgery	0.47	0.076
	Number of affected disk	0.0045	0.87
	BMI	0.45	0.093
Preoperative outcomes	Preoperative VAS	0.38	0.16
	Preoperative ODI	0.30	0.27

Top five spatiotemporal measurements with the largest absolute Spearman correlation coefficients (*r*) are listed. All clinical variables, including the functional outcomes that were collected preoperatively, are also listed

**Fig. 5:**
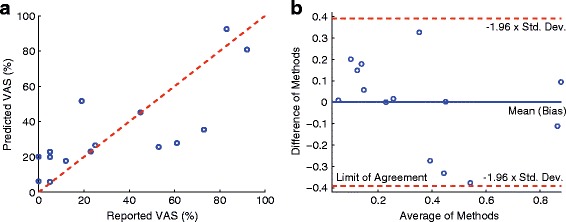
**a** *Scatter plot* between the reported VAS and predicted VAS scores, where the Spearman correlation coefficient *r* was 0.83, *p*<1.28×10^−4^, and RMSE of 0.19. **b** Bland-Altman plot where the bias was −4.22×10^−6^ and the limit of agreement was 0.39

**Fig. 6:**
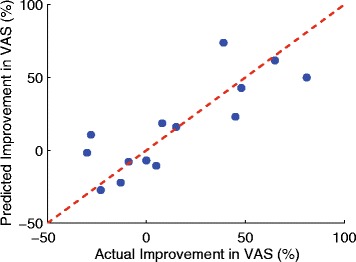
*Scatter plot* between the actual and predicted improvement in VAS after surgical operation. The Spearman correlation coefficient *r* was 0.82, *p*<2.58×10^−4^, and RMSE of 0.20

Figure 5 shows the scatter and Bland-Altman plots when two predictors were used to predict the postoperative VAS score based on multivariate linear regression technique. The best correlation was achieved when CrossCorr-P_2_ and AutoCorr-P_3_-Min were employed, which together produced *r*=0.83, *p*<1.28×10^−4^, and RMSE of 0.19. The bias and limit of agreement of the Bland-Altman plot were −4.22×10^−16^ and 0.39 respectively. Figure 6 shows the statistically significant correlation between the predicted and actual improvement of VAS after surgical operation. The RMSE was 0.20 and the Spearman correlation coefficient *r* was 0.82 with *p*<2.58×10^−4^. The unbalanced gender distribution did not show a statistically significant difference in the estimation error (*t*-test, *p*<0.33).

## Discussion

This pilot cohort study investigated the use of gait measurements derived from a pair of sensorized smart-shoes to predict postoperative functional outcomes in patients with LSS. Two validated functional outcomes routinely used in the clinical setting (ODI and VAS) were considered in this work. The gait measurements derived from the smart-shoes outperformed all other clinically available variables regarding their capacity to predict the two postoperative functional outcomes. Our method provides more quantitative measurements of outcome as compared to the more qualitative results of other works [3]. Additionally, the smart-shoe is non-invasive, inexpensive, and easy-to-use, and the 10 m SPWT takes approximately six minutes to complete. The reported results support our hypothesis that this system has great potential to provide a more accurate prediction of postoperative functional outcomes in LSS patients as compared to other predictors that have been studied and utilized to date.

Walking ability has been investigated as a predictor in LSS patients in a relatively small number of studies. Sigmundsson et al. reported that the self-estimated walking distance can be a predictor for satisfaction with operative outcomes [3]. Furthermore, preoperative walking capacity was found to be a good predictor of satisfaction with postoperative walking capacity after surgical intervention [7]. Conway et al. conducted SPWT and motorized treadmill test (MTT) [14], asking LSS patients to walk on a level ground or treadmill at a self-paced speed until they voluntarily stopped due to worsening of LSS-related symptoms or until they reached the predefined maximum time duration of 30 min. They reported a significant correlation (*p*<0.01) between the walking distance of the SPWT and the ODI score with a Pearson correlation coefficient of −0.60. Rainville et al. compared the changes in walking time and distance against the changes in ODI score [15]. A significant correlation (*p*<0.05) was reported between the walking time using the MTT and the ODI score with a Pearson correlations coefficient of 0.48. Similarly, Tomkins-Lane et al. reported a significant correlation (*p*<0.01) between the changes in walking capacity from the SPWT and changes in the ODI score with a Spearman coefficient of −0.70 [16]. However, the aforementioned works focus on measuring walking capacity, which require patients to walk up to 30 min for each test. This time requirement may limit the use of these tests in the clinical setting, notwithstanding the burden placed on patients to perform such extensive testing. Comparatively, the test proposed in this study requires a total of 40 m walking distance (10 m walking test repeated four times) and takes approximately 6 min to complete, which makes it more accessible in the clinical setting and likely more appealing to patients. Furthermore, the spatiotemporal gait characteristics provided by the smart-shoes, such as the ability to fine control the distribution of the body weight during walking, may provide more comprehensive interpretation of the LSS patient’s functional level following decompression surgery as compared to the relatively uni-dimensional evaluations of walking capacity.

Table 3 summarizes the results for predicting the postoperative ODI scores. It is noteworthy that the ODI scores collected preoperatively did not show statistical significance to the postoperative ODI scores (*p*<0.21). This may be explained by operative subjects improving non-linearly compared to their preoperative status or may be because of the response shift of the ODI; ODI is known to suffer from the response shift after surgical intervention in lumbar spinal cord disorder patients [26]. Thus, accurate prediction requires additional information, which motivated our study. None of the clinical variables investigated in this study showed significant correlation to the postoperative ODI score. The results in Table 3 reveal that the top five gait measurements with the largest (absolute) Spearman correlation coefficient contain gait information collected from P_2_ (near the heel) and/or P_3_ (mid-lateral plantar). For example, StdTime-P_2_P_3_-Max showed a positive correlation to the postoperative ODI. This implies that patients with superior functional condition (low ODI score) showed more consistent weight shifting from P_2_ to P_3_, i.e. more consistent walking pattern. Moreover, the length of time taken to shift their weight from P_2_ to P_3_ (MeanTime-P_2_P_3_-Mean) was shorter in functional patients, and thus the cumulative pressure applied to P_2_ was smaller (SumMag-P_2_-Min).This also implies that patients with higher ODI score (non-functional patients) took longer time to shift from P_2_ to P_3_, which can be explained by the sensory deficit and pain inherent to LSS resulting in a less fluid walking pattern. Patients with low ODI score also showed consistent pressure pattern at P_2_ for both limbs (AutoCorr-P_2_-Mean) and across the two limbs (CrossCorr-P_2_) during walking. When multiple predictors were considered to predict the postoperative ODI, the StdTime-P_2_P_3_-Max and SymIndex yielded the strongest (and statistically significant) correlation (*p*< 5.32 ×10^−4^). It is noteworthy that SymIndex was not one of the measurements that produced the best correlation when considered for the univariate analysis. The most likely reason that StdTime-P_2_P_3_-Mean were combined with SymIndex rather than one of the top five measurements shown in Table 3 is that the top five measurements quantify similar gait characteristics (as most of them were derived from P_2_ and/or P_3_), and thus the maximum information gain was achieved when StdTime-P_2_P_3_-Mean was combined with SymIndex, which provides different gait characteristics [27]. The predicted improvement computed by subtracting the predicted postoperative ODI score from the preoperative ODI score also showed a strong correlation compared to the actual improvement with RMSE of 0.13, *r*=0.93, and *p*<3.82×10^−7^. Thus, the ability of the smart-shoes to reliably predict changes in ODI in response to surgery based on preoperative data makes this technology a valuable tool for identifying which patients will derive the greatest benefit from surgical intervention. This will help the decision making process for both clinicians and patients when considering surgical versus medical management of LSS, since it can provide a reasonable prediction for the symptomatic improvement that a surgical intervention can reliably provide. Table 4 summarizes the predictability of the gait measurements and clinical variables for the postoperative VAS scores. None of the clinical variables showed clinically significant correlation to the postoperative VAS. However, the smoking status and the number of previous spine surgeries showed near significant correlations (*p*<0.089 for the smoking status and *p*<0.076 for the number of surgeries). This agrees with previous findings that non-smoking status [10, 11] and fewer previous surgeries [13] have a predictive influence for improved functional level and leg pain relief after surgical intervention. The VAS score that was collected preoperatively did not show significance to the postoperative VAS, which supports that predicting the improvement in pain level after surgery requires additional clinical information (other than just preoperative VAS score). Table 4 shows that the top five gait measurements with the most significant correlations measure similar gait characteristics: consistency of weight distribution during gait. However, unlike the predictors of the ODI, the predictors of the VAS included gait parameters that were extracted from different landmarks of the plantar, e.g., P_1_, P_2_, and P_5_. AutoCorr-P_2_-Mean, CrossCorr-P_2_, and AutoCorr-P_5_-Min quantified the consistency of weight distribution at P_2_ and P_5_ (or walking pattern in general). Furthermore, patients with less postoperative pain had a shorter time to shift the body weight from P_1_ and P_2_ (MeanTime-P_1_P_2_-Max) and smaller SumMag-P_2_-Min. We believe that these results resemble the results of the ODI analysis in that patients with less perceived pain were more apt to shift their weight rapidly and homogeneously to the whole plantar foot to stabilize themselves during walking. Whereas patients with higher perceived pain were more hesitant to shift their weight between limbs for fear of causing discomfort and/or instability. When more than one predictor was used to predict the postoperative VAS, CrossCorr-P_2_ and AutoCorr-P_3_-Min produced the highest correlation (*r*=0.83, *p*<1.28×10^−4^, and RMSE of 0.19). Again, one of the top individual predictors (CrossCorr-P_2_) was combined with AutoCorr-P_3_-Min, which was not included in the top five individual predictors in Table 4, i.e. a predictor that provides different dimension of gait characteristics [27]. The predicted improvement in VAS also showed a significant correlation compared to the actual improvement. Figure 6 illustrates the relationship that yielded the RMSE of 0.20, *r*=0.82, and *p*<2.58×10^−4^. It is noteworthy that, although the prediction results of postoperative score and the level of improvement in VAS were statistically significant, the results of the ODI were more significant and accurate. This agrees with prior studies that have found the ODI to be a more sensitive prognosticator as compared to the VAS score regarding surgical outcome in LSS patients [28].

The work introduced in this paper has some limitations. All patients who participated in this study were operated on by a single neurosurgeon (DCL). Thus, the predictors found in this work may vary from those found in patient populations of other surgeons. Additionally, the number of subject participants is relatively small. Thus, the identification of predictors in a large population needs to be verified, and the statistical results reported herein (e.g., the effect of the unbalanced gender distribution) may not be generalized. The research team is continuing to collect data from LSS patients and future studies will address these issues. Some factors that were previously found to have prognostic value, such as depression and psychiatric illness [3, 6], were not included in this study and their utility as predictors of outcome cannot yet be compared to the smart-shoe data. Additionally, patients were reevaluated three months after their scheduled surgeries in order to obtain postoperative ODI and VAS results. Amundsen et al. reported that while most patients experienced relief of pain approximately three months after surgical intervention, pain levels in these patients would continue to decrease over years [29]. Therefore, longer-term follow-up may be necessary to discover predictors of more permanent postoperative clinical outcomes. It is worth noting, however, that Atlas et al. found that a patient’s baseline postoperative functional level was reached by three months after the surgical intervention, i.e. patients function level did not improve much after three months postoperatively [30]. This supports our belief that the reported predictors for our three-month follow-up study should provide insights into the long-term outcomes of our LSS patient population.

## Conclusion

This paper introduced a method that can be easily implemented in the clinical setting to predict postoperative functional outcomes and the expected benefit of the surgical intervention in LSS patients, based on the preoperative gait analyses and clinical data. This method analyzes walking characteristics of LSS patients using a pair of sensorized smart-shoes and a series of 10 m SPWTs. A total of 39 gait parameters extracted from the preoperative walking tests and ten clinical variables were considered as possible postoperative predictors. Two clinical outcomes, i.e. ODI and predominant pain via VAS, were used to establish the postoperative functional level. It has been demonstrated that the gait parameters extracted from the smart-shoes can make statistically significant predictions of the postoperative (and the expected improvement in) ODI and VAS, thereby assisting clinicians in identifying which patient population can benefit from the surgical intervention. It was also shown that the gait parameters improved prediction accuracy as compared to the clinical variables that have been previously utilized as postoperative outcome predictors. The smart-shoe system is non-invasive, inexpensive, easy-to-use, and the walking test takes approximately six minutes to complete. Thus, the smart-shoe system is ideally suited for preoperative evaluation in the clinical setting. We believe that our findings enable new clinical and research opportunities for investigating prognostic factors and optimizing patient selection for LSS surgery.

## Additional file

Additional file 1 Appendix. (PDF 67 kb)

## Abbreviations

BMI: body mass index
LSS: lumbar spinal stenosis
MRI: magnetic resonance imaging
MTT: motorized treadmill test
ODI: Oswestry disability index
RMSE: root mean squared error
SPWT: self-paced walking test
VAS: Visual analogue scale
