# Supplementary material for: Identifying predictors for postoperative clinical outcome in lumbar spinal stenosis patients using smart-shoe technology
Source: J Neuroeng Rehabil. 2017 Jul 18;14:77. doi: 10.1186/s12984-017-0288-0 (PMC5516369; doi:10.1186/s12984-017-0288-0)
Supplement: Additional file 1 — Appendix. (PDF 67 kb) [file 12984_2017_288_MOESM1_ESM.pdf]

Appendix

This section provides detailed information for all the spatiotemporal measurements that were extracted from the smart-shoes in order to estimate the outcome measures. MeanGCT and StdDevGCT represent the mean and standard deviation of the gait cycle time (GCT) that were computed using P_1, i.e. heel-strikes. MeanTime-$\mathrm{P}_i\mathrm{P}_j$ and StdDevTime-$\mathrm{P}_i\mathrm{P}_j$ represent the mean and standard deviation of the time difference between the peaks of P_i and P_j, respectively. Note that MeanTime-$\mathrm{P}_1\mathrm{P}_5$ equivalently represents the stance time. MeanSSR and StdDevSSR computed the mean and standard deviation of the stance-to-stride ratio. All the aforementioned time-related measurements were normalized to the shoe size (i.e. length of the shoe) in order to remove distance-dependent variability.

AutoCorr-P_i computed the maximum auto-correlation of the time series of P_i. CrossCorr-$\mathrm{P}_i\mathrm{P}_j$ computed the maximum cross-correlation between the time series of P_i and P_j. These two measurements were computed per 10m walk and averaged out of the four walks. SumMag-P_i, MeanMag-P_i, StdDevMag-P_i represent the sum, mean, and standard deviation of the amplitudes of P_i, respectively. These three parameters were computed per gait cycle on each foot, and averaged over the gait cycles that were obtained from the four 10m walks. All the aforementioned parameters were calculated independently from the two feet, and the minimum, maximum, and mean of the two values were considered in order to emphasize the unilateral (minimum and maximum), and bilateral (mean) characteristics of the motor symptoms in LSS patients.

This study also included some gait measurements that were computed as a function of sensor data from the two shoes. The symmetry index of gait, SymIndex, investigated the bilateral symmetry between the two limbs: SymIndex $= (T_R - T_L)/\frac{1}{2}(T_R + T_L)$, where T_R and T_L represent the average stride time for left and right feet, respectively. CrossCorr-P_i computed the maximum cross-correlation of the time series of P_i of the left and right shoes.
